# Pre-arthritic coronal plane alignment predicted by the arithmetic hip-knee-ankle angle (aHKA) and the Flexion Extension Balancing Algorithm (FEBA) for Primary Total Knee Arthroplasty (TKA)

**DOI:** 10.1016/j.jor.2024.11.024

**Published:** 2024-11-26

**Authors:** Tristan Pillay, Anthony O'Neill, Philip Hay, Michael McAuliffe

**Affiliations:** a Townsville University Hospital, Queensland Health, Townsville, QLD, 4814, Australia; b James Cook University, Townsville, QLD, 4812, Australia; c The Mater Public and Private Hospital, South Brisbane, QLD, 4101, Australia; d Brisbane Private Hospital, Brisbane City, QLD, 4000, Australia; e Surgical Outcomes Research Centre (SOuRCe), Camperdown, NSW, 2050, Australia; f Ipswich General Hospital, Queensland Health, Ipswich, QLD, 4305, Australia; g Queensland University of Technology, Brisbane, QLD, 4000, Australia; h The Mater Private Hospital, Springfield Lakes, QLD, 4300, Australia; i St Andrew's Ipswich Private Hospital, Ipswich, QLD, 4305, Australia

## Abstract

**Background:**

Given the relative rate of dissatisfaction following total knee arthroplasty (TKA) and the need to further improve outcomes for all patients, various surgical methods have been developed that aim to restore the pre-arthritic alignment of the knee and lower limb. Common to these methodologies is a need to determine the pre-arthritic alignment of the knee and limb, thus producing defined targets for surgery. The aim of this paper was to compare the predicted pre-arthritic constitutional alignment of knee and lower limb calculated by the Flexion Extension Balancing Algorithm (FEBA) and the arithmetic HKA (aHKA) methods. The aHKA has been proposed as a means of accurately estimating the constitutional alignment of a knee and lower limb.

**Methods:**

We calculated the proposed pre-arthritic alignment of 78 knees immediately prior to TKA surgery based on calculations using both methods. The results produced by the FEBA planning system (fHKA) were compared to the coronal plane alignment proposed by the aHKA process.

**Results:**

No significant difference was demonstrated between the pre-arthritic alignments calculated by the two methods. The mean aHKA was −1.5° (SD 3.38°; range −7.9°–6.9°) and the mean fHKA was −1.1° (SD 2.96°; range −8.1°–7.5°). The mean angular difference between the methods was 0.4° ±1.94; p = 0.146. The two methods produced alignment measurements with a strong positive correlation r = 0.82 p < 0.0001; R^2^ = 0.674.

**Conclusions:**

There is a high correlation between the proposed pre-arthritic knee alignments when comparing the FEBA and arithmetic HKA methods. The pre-arthritic alignment of the knee is difficult to know with certainty. The use of both calculation methods will deliver a potential target zone for TKA knee alignment that makes use of all residual anatomy.

## 1 Introduction

Primary Total Knee Arthroplasty (TKA) is a common and increasingly utilised procedure. In 2021 approximately 60,000 and 700, 000 Primary TKAs were undertaken in Australia and the USA respectively.^1^^,^^2^ The cost of TKA surgery, in Australia alone, is projected to be $5.32 billion by 2030.^3^ Multiple studies delineate a cohort of patients who report dissatisfaction with TKA. This dissatisfaction rate is persistent. In 2021 patient reported outcome measures (PROMS) from the Australian Orthopaedic Association National Joint Replacement Registry reported an 84.5 % dissatisfaction rate which is similar to the rate reported by the Swedish Registry 9 years prior.^1^^,^^4^^,^^5^

In an effort to improve primary TKA outcomes multiple authors advocate using the procedure to restore the knee anatomy to its pre-arthritic alignment. This concept is based on the premise that restoration of native anatomy will personalise knee biomechanics and lessen any distorting forces on the peri-articular soft tissues of the knee thus optimizing patient outcomes in that regard.^6^^,^^7^ Multiple surgical methods have been proposed for achieving a more individualised prosthesis position, these include kinematic, restricted kinematic, reverse kinematic and anatomic alignment theories.^8^, ^9^, ^10^, ^11^ The underlying challenge common to all these surgical methods is how best to determine the pre-arthritic alignment of the knee and overall lower limb.

The Flexion Extension Balancing Algorithm (FEBA) system was first published in 2015.^12^^,^^13^ It is a pre-operative planning method for TKA. It is designed to formulate a surgical plan that reproduces multiple parameters including the pre-arthritic coronal plane alignment of the extended knee, the reproduction of the medial joint line and the attainment of balanced flexion and extension gaps. Its method of calculating the pre-arthritic position of the limb is based on long-leg alignment radiographs and the residual cartilage, measured by MRI, in the less diseased tibiofemoral compartment.

The arithmetic hip-knee-ankle angle (aHKA) algorithm, first published in 2021, is an alternative method used to predict the constitutional alignment and phenotype of the arthritic knee once osteoarthritis has developed.^14^ It has been validated against the normal contralateral knee through a matched-pairs radiological study.^15^ This study demonstrated a modest mean difference in overall alignment parameters between the groups. However, it also recognized absolute differences ranging between 3 and 4.8° of varus and valgus respectively for the studied pairs.^15^ Similar to the FEBA method, the aHKA algorithm is based on the evaluation of long-leg alignment radiographs and is therefore well-suited for a comparison of predicted outcomes.^15^

The aim of this study was to assess comparability in the predicted coronal plane constitutional alignment using the FEBA (fHKA) method compared to the arithmetic HKA (aHKA) method.

## 2 Materials and methods

### 2.1 Study design

Retrospective review of routinely prospectively collected clinical data was undertaken following ethics approval from an independent institutional review board for 78 TKA cases pre-operatively planned with the FEBA method.

### 2.2 Study group

All patients were planned to undergo a primary total knee arthroplasty using the Journey II BCS prosthesis (Smith & Nephew, Memphis, USA) for a primary indication of osteoarthritis. The study cohort consisted of 45 (58 %) women and 33 (42 %) men.

As per FEBA clinical boundaries, patients were included if they had a pre-operative deformity ≤15° varus or 10° valgus. Patients with a history of prior knee trauma distorting bony anatomy, extra-articular deformities of the femur or tibia, a prior femoral or tibial osteotomy, evidence of significant bi-condylar cartilage loss or gross ligamentous deficiency were excluded.

### 2.3 Imaging technique

Weight-bearing long-leg radiographs and magnetic resonance imaging (MRI) scans were taken according to a Patient-Specific Instrumentation (PSI) protocol (Visionaire, Smith & Nephew, Memphis, USA).

Radiographs were reviewed for image quality and rotation by an imaging technician in accordance with the standard PSI development process. A physical scaling marker was included adjacent to the operative knee during imaging to permit 1:1 scaling of the digital radiographic images using the pre-operative planning software.

The MRI scans were undertaken at two sites using a Siemens Magnetom Skyra (3T), and a Siemens Magnetom Lumina (3T) MRI scanner. Patients were positioned supine with their operative knee in extension and stabilized in a knee holder. Images were acquired using a sagittal scan sequence and 2 mm slice thickness with the joint space centred in the field of view.

### 2.4 Radiographic analysis technique

Radiographs were templated according to the methods described by Paley.^16^ The femoral mechanical axis (MA) was determined from the centre of the femoral head to the centre of the distal femur at the level of the knee and the tibial MA determined using the midpoint of the tibia at the level of the knee to the centre of the ankle. The mechanical hip-knee ankle angle (mHKA) was then the angle subtended by the MA of the femur and MA of the tibia.

The lateral distal femoral angle (LDFA) and medial proximal tibial angle (MPTA) were assessed following the method described by Griffiths-Jones et al..^14^ Subsequently, the LDFA and MPTA were used to calculate the arithmetic HKA (aHKA) and joint-line obliquity (JLO) to determine the native coronal plane alignment of the knee (CPAK) phenotype.^17^ As per the described technique we refer to varus deformity as a negative number and valgus deformity as a positive number.

aHKA = MPTA - LDFA^17^

JLO = MPTA + LDFA^17^

### 2.5 MRI analysis technique

MRI scans were imported into a DICOM segmentation and viewing software (VSeg, Siemens Healthcare, Erlangen, Germany) for analysis. A method was developed using the VSeg program to undertake manual measurement of the distal femoral and proximal tibial cartilage thickness of the compartment least affected by osteoarthritis. The most distal point of the femoral condyle was identified in the sagittal plane. Subsequently, this point was identified in the coronal plane to confirm the location was central to the respective condyle. The point-to-point measurement tool was then used to determine the cartilage thickness to the articular surface of the respective distal femoral condyle and proximal tibial plateau. The distal femoral cartilage (DFC) and proximal tibial cartilage (PTC) measurements were incorporated into the pre-operative planning following the FEBA method.

All imaging measurements were undertaken by a biomedical engineer independently of the study authors. The engineer documented the parameters as part of the standard pre-operative planning process, and they were blinded to the measurements being used for a clinical study.

### 2.6 The FEBA planning method

The FEBA coronal-plane correction angle (CCA) is defined as the angle available for correction of the pre-operative deformity measured between the articular surfaces of the less diseased tibiofemoral compartment.

To determine the CCA, the standing long-leg radiographs were scaled 1:1 using the pre-operative planning software. Splines were created following the articular surfaces of the distal femoral condyle and proximal tibial plateau and offset by the DFC and PTC measurements obtained from the MRI analysis. The CCA was measured as the angular difference between the most distal point of the DFC spline and the corresponding point of the PTC spline taken about the proximal tibial MA point.

**Fig. 1:**
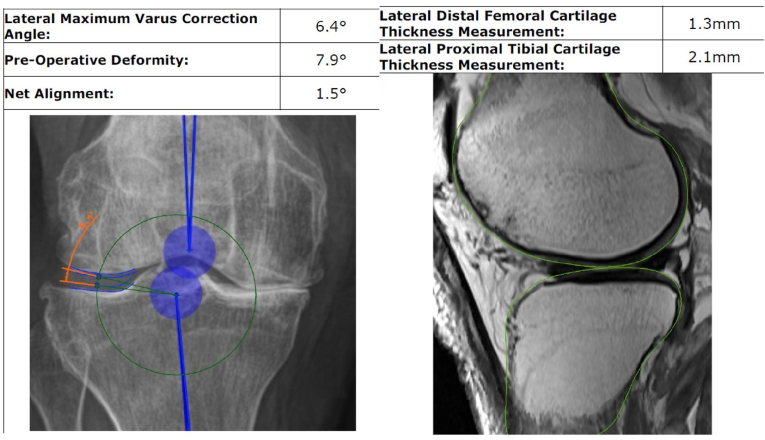
FEBA coronal plane position calculation. The cartilage in the less diseased compartment is measured. The correction angle is then calculated based on the available space allowing a determination of the net alignment.

The pre-arthritic coronal alignment, or FEBA HKA (fHKA) is predicted by the addition of the CCA to the mHKA. The mHKA is a negative value for varus alignment and positive for valgus alignment. The CCA is assigned a positive value for a varus deformity and a negative value for a valgus deformity as shown in Fig. 1 fHKA = mHKA + CCA.

### 2.7 Statistical analysis

Macdessi et al.^15^ calculated a minimum of 47 radiographs were required to detect a maximum difference of 1.5° between matched pairs with a power of 80 %, and statistical significance of 5 %. Our sample size was based on prior data indicating that the difference in the response of matched pairs is normally distributed with a standard deviation 2.50. If the true difference in the mean response of matched pairs is 1°, we needed 68 subjects to be able to reject the null hypothesis that this response difference is zero with probability (power) 0.90. The Type I error probability associated with this test of this null hypothesis is 0.05.

Statistical analysis was undertaken using Microsoft Excel and R-Studio. Descriptive statistics were used for calculation of means, SD, and 95 % confidence intervals. Paired t-tests were used for comparison of means between radiological alignment parameters. Normal distribution of data was confirmed by analysing histograms of continuous variables. Pearson's r (r) and the coefficient of determination (R^2^) were calculated comparing aHKA and fHKA.

Independent two-tailed t-tests were used for analysis of mean differences in deformities greater than and less than or equal to 8°, as well as differences between sexes. Significance was set with a p-value <0.05.

## 3 Results

The demographics of the study cohort are defined in Table 1. The average age was 68 years (SD 7.5 years; range 55–86 years).

**Table 1:** Demographics.

Variable	N (%)	Mean ± SD	Range
Age (years)	78	68 ± 7.5	55 to 86
BMI (kg/m^2^)	78	32.4 ± 6.1	20.1 to 48.3
Sex			
Male	33 (42 %)	–	–
Female	45 (58 %)	–	–
Operative Side			
Left	36 (46 %)	–	–
Right	42 (54 %)	–	–
Pre-Operative Deformity			
Varus	65 (84 %)	−7.2 ± 3.4	−0.3 to −14.7
Valgus	13 (16 %)	4.5 ± 3.5	0.8 to 9.9

The deformity range of the cohort was limited to pre-operative deformities between −15° varus and 10° valgus as per FEBA criteria. The majority of cases were pre-operative varus deformities.

The mean mHKA of the arthritic knee was −5.3° (SD 5.43°; range −14.7 to 9.9°) with a significant difference between male knees (mean −7.4°; SD 4.49°) and female knees (mean −3.7°; SD 5.55°), demonstrating greater varus deformity on average for male knees (Table 2).

**Table 2:** Radiological parameters between sexes.

Variable	All Mean ± SD (n = 78)	Male Mean ± SD (n = 33)	Female Mean ± SD (n = 45)	p-value between sexes
mHKA (°)	−5.3 ± 5.43	−7.4 ± 4.49	−3.7 ± 5.55	0.002^a^
LDFA (°)	87.8 ± 1.89	88.3 ± 1.70	87.5 ± 1.95	0.053
MPTA (°)	86.3 ± 2.51	85.3 ± 2.50	87.0 ± 2.30	0.004^a^

a Indicates significant p-value.

### 3.1 Primary outcome

The mean aHKA and fHKA demonstrated statistically significant differences between male knees and female knees, with the constitutional alignment predicted using both methods demonstrating greater varus alignment on average for male knees (mean aHKA of −3.0°; mean fHKA of −2.2°) than female knees (mean aHKA of −0.5°; mean fHKA of −0.4°). The mean difference between the aHKA and fHKA was 0.4° (SD 1.94°) and was not statistically significant when compared between sexes (p = 0.146).

**Fig. 2:**
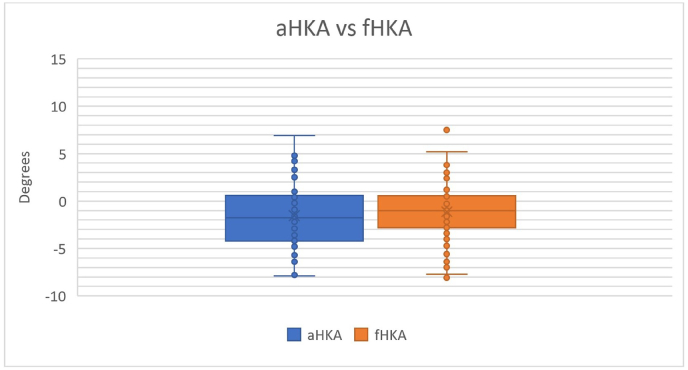
Arithmetic Hip-Knee-Ankle Angle (aHKA) vs Derived FEBA Hip-Knee-Ankle Angle (fHKA).

Knees with pre-operative deformities greater than 8° (n = 27) had a mean difference of 0.7° (SD 1.85°; range −3.9°–3.4°) between the fHKA and aHKA compared to those with deformities less than or equal to 8° with a mean difference of 0.3° (SD 1.98°; range −3.9°–4.9°)(Fig. 2). However, these differences were not statistically significant (p = 0.355) when comparing the two groups.

Fig. 2 demonstrates the spread of the calculated pre-disease HKA of the study cohort by the FEBA and CPAK methods.

The mean aHKA was −1.5° (SD 3.38°; range −7.9°–6.9°) and the mean fHKA was −1.1° (SD 2.96°; range −8.1°–7.5°). Results showed no significant difference between fHKA and aHKA (t = 1.85 with 77 degrees of freedom; p = 0.064), with a mean angular difference of 0.4° (95 % CI −0.02° to 0.85°; paired *t*-test) (Table 3, Table 4).

**Table 3:** aHKA and fHKA differences between sexes.

Variable	All Mean ± SD (n = 78)	Male Mean ± SD (n = 33)	Female Mean ± SD (n = 45)	p-value Male vs Female
aHKA (°)	−1.5 ± 3.38	−3.0 ± 3.07	−0.5 ± 3.24	0.001^a^
fHKA (°)	−1.1 ± 2.96	−2.2 ± 2.82	−0.4 ± 2.85	0.007^a^
Mean difference between aHKA and fHKA (°)	0.4 ± 1.94	0.8 ± 1.80	0.1 ± 2.00	0.146

a Indicates significant p-value.

**Table 4:** aHKA and fHKA differences between pre-operative deformity.

Group	N (%)	Mean difference in fHKA and aHKA ± SD (°)	Range (°)	p-value
Pre-operative deformity ≤8°	51 (65 %)	0.3 ± 1.98	−3.9 to 4.9	–
Pre-operative deformity >8°	27 (35 %)	0.7 ± 1.85	−3.9 to 3.4	–
Difference in means between groups	–	−0.4	–	0.355

**Fig. 3:**
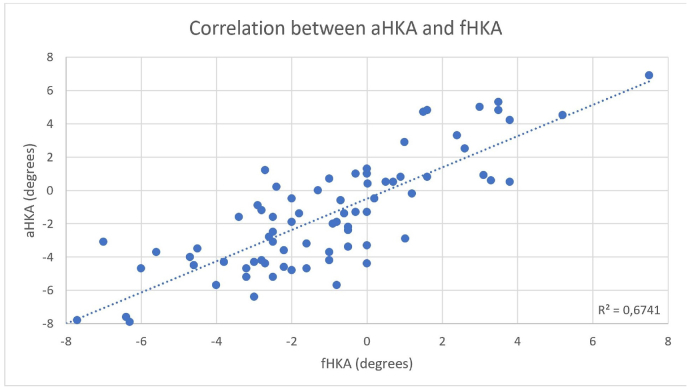
Correlation between aHKA and fHKA.

In addition, fHKA and aHKA were strongly positively correlated (r = 0.82, p < 0.0001; R^2^ = 0.674) with 67 % of the variability in aHKA explained by the variability in fHKA (Fig. 3).

### 3.2 Secondary outcomes

Cartilage thickness in the less diseased compartment demonstrated significant variation as displayed in Table 5. The mean proximal tibial cartilage thickness was 2.3 mm (SD 0.80 mm; range 0.5–4.4 mm) and the mean distal femoral cartilage thickness was 1.8 mm (SD 0.51 mm; range 0.4–3.7 mm).

**Table 5:** Cartilage thickness in the less diseased compartment.

Variable	N	Mean ± SD	Range
Proximal Tibial Cartilage Thickness (mm)	78	2.3 ± 0.80	0.5 to 4.4
Distal Femoral Cartilage Thickness (mm)	78	1.8 ± 0.51	0.4 to 3.7
Combined Cartilage Thickness (mm)	78	4.1 ± 1.16	1.0 to 7.0

## 4 Discussion

Our results provide little or no evidence to reject the null hypothesis that there is no significant difference between the pre-arthritic alignment calculated by the FEBA and aHKA methodologies given the mean angular difference between the methods was 0.4° ±1.94; p = 0.146 compared to our threshold of 1°.

Whilst the null hypothesis appears valid, the clinical interpretation of these findings is important and there are several factors that may have influenced the correlation between the two calculated values.

From a clinical perspective the aHKA is not purported to demonstrate the pre-arthritic position of an individual knee. In a matched pairs study, there was a small mean difference and a high correlation between the mechanical HKA of normal knees and aHKA of the paired osteoarthritic knee. This finding was interpreted by the authors as validating that from an overall cohort perspective the aHKA method was reasonable for reproducing the pre-disease position of the knee. The mean difference in alignment cannot be applied to an individual knee with certainty. In the same study the authors also reported clinically relevant differences between the aHKA of the arthritic limb and the mechanical HKA of the matched normal limb of between 3 and 4.8° of varus and valgus respectively.^15^ Therefore, prior literature reports the aHKA as providing a reasonable but not absolute approximation of the pre-disease position of the limb. From a surgical perspective an alignment target that imparts maximum information and is as precise as possible for a specific individual is important for surgical planning and execution. It is for this reason that we wanted to compare the two calculated values and consider how they might contribute to surgical planning.

Firstly, we would acknowledge the limitations of our study and the potential impact on our results. The quality of the long-leg radiographs and MRI scans may have been variable, thus impacting the calculated values. The images were ultimately utilised to produce 3D patient specific surgical guides and were independently deemed to have met the required standard for this manufacturing process meaning radiographic investigational quality is unlikely to have affected the study results. The measurements of the various anatomical landmarks were subject to single observer measurement which is a potential source of error. Mitigating this, is the fact that these measurements were collected independently by a trained biomedical engineer who was blinded to the measurements being used for a clinical study.

The osteoarthritic process will potentially have had unequal effects on the residual bone and cartilage anatomy, this would differentially affect the calculated pre-arthritic alignment values and the subsequent correlation. Given that the measured cartilage thickness and bony angular measurements around the knee were similar to those seen in healthy subjects this is not likely to have significantly altered the results.

The fHKA utilises long-leg radiographs with the addition of consideration of the cartilage thickness. Normal distal femoral cartilage thickness has been noted on the medial and lateral sides to respectively have mean values of 2.13 and 1.99 mm, whilst the range in normal values varies between 0.53 and 3.44 mm^18^. These results are very similar to the values in our subjects with a mean distal femoral thickness in the less diseased compartment of 1.80 mm with a range 0.40–3.70 mm. Furthermore, we had a predominance of females in our cohort who are known to have thinner normal femoral cartilage.^18^ Cadaveric studies have documented normal proximal tibial cartilage thickness on average to be between 2.07 and 2.98 mm which is similar to our mean proximal tibial cartilage thickness of 2.30 ± 0.80 mm^19^. The range in cartilage thickness we documented is typical of normal anatomical variation. This means that the FEBA calculation is not likely to have been significantly impacted by osteoarthritis causing marked changes in the cartilage thickness in the less diseased compartment.

Bellemans et al. found a mechanical HKA in young healthy subjects of −1.33° ±2.34, in that study, females were noted to have significantly less varus alignment with a mechanical HKA of −0.79° ±2.1^19^. The fHKA of −1.1° ±2.96 closely approximates the alignment value for young healthy subjects particularly given the predominance of females in our study cohort. Similarly, the bone parameters calculated in our cohort closely matched those in the study of Bellemans et al..^19^ Bellemans reported a mean LDFA of 87.90° ±1.74 and an MPTA of 87.40° ±2.07 compared to respective values of 87.80° ±1.89 and 86.30° ±2.51 in our study cohort. Therefore, the bony parameters and subsequently calculated pre-arthritic limb alignment are both consistent with measurements seen in healthy subjects and prior studies. This would support the relevance of our aHKA results and mean that the two calculation methods can be reasonably utilised and compared.

There are multiple reasons why a variation between the two calculated values may arise. Both methods rely on the use of residual anatomy that is believed to be minimally altered by the osteoarthritic process. The aHKA method was introduced as a means of determining the constitutional alignment of the limb once the alignment has been altered by osteoarthritis. It utilises the interaction between the MPTA and the LDFA.^15^ It relies on the absence of arthritic bone loss in the central compartment contact points. Additionally, it is important to appreciate that the aHKA is only an approximation of constitutional alignment and adopts the notion that the distal femoral and proximal tibial joint lines are parallel in the normal knee rather than considering the joint line convergence angle.^15^ Therefore, other methods of determining pre-disease alignment of the limb are likely to be valuable as the aHKA does not represent an individualised “gold” standard measurement.

The mean contribution of joint line convergence to knee and limb alignment is 0.51° ±1.05 ^14^. The FEBA methodology utilises residual cartilage interaction potentially reproducing native alignment including native joint line convergence. The mean angular difference between the variables was 0.40° ±1.94 which approximates the expected input of joint line convergence. This difference in the methodologies is likely to account in part for differences in the calculated outcomes. We have documented significant variability in cartilage thickness which is consistent with normal anatomical variation. The aHKA method does not account for cartilage thickness which will further contribute to differences in the values calculated for the pre-arthritic limb position.

The mean angular difference between the methods was 0.40° ±1.94; p = 0.146. The two methods produced alignment measurements with a strong positive correlation r = 0.82 p < 0.0001; R^2^ = 0.674. Given the previous validation of the aHKA against matched normal radiographs our results suggest that both methods are highly likely to approximate the pre-arthritic position of the limb, without either being interpreted as being absolute.^14^^,^^15^ As noted, the aHKA method does not claim to demonstrate the absolute pre-disease position of an individual knee, the FEBA alignment calculation should be interpreted in a similar manner. Whilst there is no significant difference between the methods when considering the mean angular difference, it is also important to note that the two methods did differ in their calculations of the pre-arthritic position of the knee to a degree that in many instances would be clinically significant. The range in the difference in the calculated positions was up to −3.9° and 4.9°. This range is similar to that seen in the matched pairs aHKA study.^15^ Therefore, when considering the pre-arthritic position of the limb the use of both methods is likely to be useful for producing a target zone for surgery that will be of greater value to the surgeon rather than simply adhering to a single alignment calculation that has not been advocated to be applied in an absolute manner.

Additionally, Macdessi et al. found greater discrepancies in measurements between the aHKA and corresponding normal radiographs for deformities over 8°.^15^ Our results demonstrated no significant difference in the mean angular difference between the two methods for deformities above and below 8°. This would suggest that the anatomy being utilised for calculating the pre-arthritic knee position is not significantly altered at this level of deformity.

**Fig. 4:**
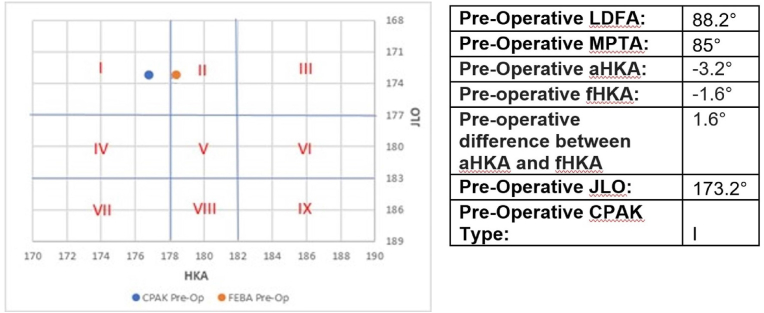
Case example of some difference identified between aHKA and fHKA in our cohort.

**Fig. 5:**
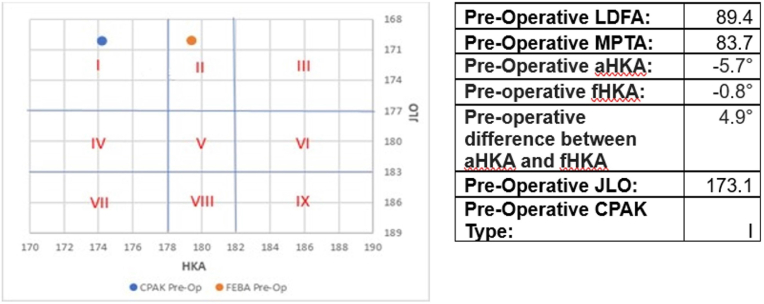
Case example with the greatest difference identified between aHKA and fHKA in our cohort.

We have documented a strong positive correlation between the two methods for calculating the pre-arthritic position of the knee. It is challenging to determine whether the OA process has caused subtle remodelling of the bone or alterations in the thickness of the cartilage in the less diseased compartment. Therefore, there is likely to be value in calculating both values as this will deliver a target zone for surgery rather than a single position. Examples of the practical application of our results to surgery are depicted in Fig. 4, Fig. 5.

Our findings have direct applicability to surgical workflows. There is limited evidence for the presence of contractures in the osteoarthritic knee particularly at deformities between 10° of valgus and 15° of varus.^18^, ^19^, ^20^, ^21^, ^22^, ^23^ As such the knee is likely to be able to attain the calculated alignment if that position is representative of the pre-arthritic disease condition. Depending on the inherent coronal plane laxity of the knee which is known to be highly variable in both the healthy and arthritic knee^18^, ^19^, ^20^, ^21^, ^22^, ^23^, ^24^, ^25^, ^26^, ^27^, ^28^ the surgeon can decide where to position the knee in the relation to the calculated aHKA and fHKA values whilst allowing for knee laxity that is commensurate with good TKA clinical outcomes.^29^, ^30^, ^31^, ^32^

## 5 Conclusion

We have demonstrated a strong positive correlation between the two different methods for calculating the pre-arthritic position of the knee providing little to no evidence to reject our null hypothesis. It is of course impossible to be absolute about the pre-arthritic alignment of the knee and lower limb. It is for this reason we propose that understanding both values is likely to produce a useful target zone for surgeons.

It is also important to understand that this analysis focusses on the coronal plane alignment of the knee in maximum extension and that the true pre-arthritic position of the limb relates to multiple positions and planes.

Future studies should focus on the further assessment of the aHKA and fHKA calculated alignment compared to contralateral knees with minimal osteoarthritic change and the relationship of the proposed alignment to subsequent clinical outcomes. Furthermore, the application of FEBA should be subjected to rigorous medium and long-term evaluation looking at both patient and prosthetic outcomes to truly validate the potential benefits of this technique.

## CRediT authorship contribution statement

**Tristan Pillay:** Conceptualization, Writing – original draft, Data curation, Visualization. **Anthony O'Neill:** Conceptualization, Methodology, Investigation, Writing – original draft. **Philip Hay:** Data curation, Formal analysis, Supervision, Writing – review & editing. **Michael McAuliffe:** Conceptualization, Methodology, Writing – original draft, Supervision.

## Patient/guardian consent

As per ethics approval from the Institutional Review Board, all participants included in this study provided the appropriate informed consent. The ethics review details are as follows:

Project ID: 2023/PID/0155 HREC ID: 2023/ETH/0066 Project Title: What is the Correlation between the Flexion and Extension Balancing Algorithm (FEBA) and the arithmetic HKA for the Predicted Pre-Arthritic Coronal Alignment in Patients Undergoing Primary Total Knee Arthroplasty for Osteoarthritis? (retrospective review of medical records).

Please do not hesitate to contact me, should you require further information.

## Funding statement

Please be advised that no additional funding was obtained for our study entitled: Pre-Arthritic Coronal Plane Alignment Predicted by the Arithmetic Hip-Knee-Ankle Angle (aHKA) and the Flexion Extension Balancing Algorithm (FEBA) for Primary Total Knee Arthroplasty (TKA).

## References

[1] (AOANJRR) AOANJRR (2022). https://aoanjrr.sahmri.com/annual-reports-2022.

[2] Fingar K.R., Stocks C., Weiss A.J., Steiner C.A. (2006).

[3] Ackerman I.N., Bohensky M.A., Zomer E. (2019/02/23 2019). The projected burden of primary total knee and hip replacement for osteoarthritis in Australia to the year 2030. BMC Muscoskel Disord.

[4] Bourne R.B., Chesworth B.M., Davis A.M., Mahomed N.N., Charron K.D. (Jan 2010). Patient satisfaction after total knee arthroplasty: who is satisfied and who is not?. Clin Orthop Relat Res.

[5] Swedish Knee Arthroplasty Register: Annual Report 2014. Lund University, Sweden2014..

[6] Lustig S., Sappey-Marinier E., Fary C., Servien E., Parratte S., Batailler C. (2021). Personalized alignment in total knee arthroplasty: current concepts. Sicot-j..

[7] Rivière C., Iranpour F., Auvinet E. (2017/11/01/2017). Alignment options for total knee arthroplasty: a systematic review. J Orthop Traumatol: Surg Res.

[8] Howell S.M., Papadopoulos S., Kuznik K.T., Hull M.L. (Oct 2013). Accurate alignment and high function after kinematically aligned TKA performed with generic instruments. Knee Surg Sports Traumatol Arthrosc : off J ESSKA.

[9] Almaawi A.M., Hutt J.R.B., Masse V., Lavigne M., Vendittoli P.A. (Jul 2017). The impact of mechanical and restricted kinematic alignment on knee anatomy in total knee arthroplasty. J Arthroplasty.

[10] Winnock de Grave P., Luyckx T., Claeys K. (Feb 2022). Higher satisfaction after total knee arthroplasty using restricted inverse kinematic alignment compared to adjusted mechanical alignment. Knee Surg Sports Traumatol Arthrosc : off J ESSKA.

[11] Hungerford D.S., Kenna R.V., Krackow K.A. (Jan 1982). The porous-coated anatomic total knee. Orthop Clin N Am.

[12] Daniel Raymond Lawson SS., Bartholomew Cavanagh Conan, Mcauliffe Michael (2015).

[13] McAuliffe M., Roe J.A., Garg G. (2016). A new method to attain a balanced total knee arthroplasty. Orthop J Sports Med.

[14] Griffiths-Jones W., Chen D.B., Harris I.A., Bellemans J., MacDessi S.J. (May 2021). Arithmetic hip-knee-ankle angle (aHKA): an algorithm for estimating constitutional lower limb alignment in the arthritic patient population. Bone Joint Open.

[15] MacDessi S.J., Griffiths-Jones W., Harris I.A., Bellemans J., Chen D.B. (Jul 2020). The arithmetic HKA (aHKA) predicts the constitutional alignment of the arthritic knee compared to the normal contralateral knee: a matched-pairs radiographic study. Bone Joint Open.

[16] Paley D. (2002).

[17] MacDessi S.J., Griffiths-Jones W., Harris I.A., Bellemans J., Chen D.B. (Feb 2021). Coronal plane alignment of the knee (CPAK) classification. Bone Joint J.

[18] Jenny J.Y. (Sep 2010). Coronal plane knee laxity measurement: is computer-assisted navigation useful?. Orthop Traumatol Surg Res : OTSR.

[19] Bellemans J., Vandenneucker H., Vanlauwe J., Victor J. (Feb 2010). The influence of coronal plane deformity on mediolateral ligament status: an observational study in varus knees. Knee Surg Sports Traumatol Arthrosc : off J ESSKA.

[20] Marcovigi A., Zambianchi F., Giorgini A., Digennaro V., Catani F. (Dec 2016). The impact of bone deformity on osteoarthritic varus knee correctability. J Arthroplasty.

[21] Hohman D.W., Nodzo S.R., Phillips M., Fitz W. (Dec 2015). The implications of mechanical alignment on soft tissue balancing in total knee arthroplasty. Knee Surg Sports Traumatol Arthrosc : off J ESSKA.

[22] McAuliffe M.J., Vakili A., Garg G., Roe J., Whitehouse S.L., Crawford R. (Sep-Dec 2017). Are varus knees contracted? Reconciling the literature. J Orthop Surg.

[23] McAuliffe M.J., Garg G., Orschulok T., Roe J., Whitehouse S.L., Crawford R. (Jan-Apr 2019). Coronal plane laxity of valgus osteoarthritic knee. J Orthop Surg.

[24] Heesterbeek P.J., Verdonschot N., Wymenga A.B. (Jan 2008). In vivo knee laxity in flexion and extension: a radiographic study in 30 older healthy subjects. Knee.

[25] Tokuhara Y., Kadoya Y., Nakagawa S., Kobayashi A., Takaoka K. (Nov 2004). The flexion gap in normal knees. An MRI study. J Bone Joint Surg Br.

[26] Okazaki K., Miura H., Matsuda S. (May 2006). Asymmetry of mediolateral laxity of the normal knee. J Orthop Sci : off Jpn Orthop Assoc.

[27] Roth J.D., Howell S.M., Hull M.L. (Oct 21 2015). Native knee laxities at 0°, 45°, and 90° of flexion and their relationship to the goal of the gap-balancing alignment method of total knee arthroplasty. J Bone Joint Surg Am Vol.

[28] Creaby M.W., Wrigley T.V., Lim B.W. (Sep 2010). Varus-valgus laxity and passive stiffness in medial knee osteoarthritis. Arthritis Care Res.

[29] Seah R.B., Yeo S.J., Chin P.L., Yew A.K., Chong H.C., Lo N.N. (Dec 2014). Evaluation of medial-lateral stability and functional outcome following total knee arthroplasty: results of a single hospital joint registry. J Arthroplasty.

[30] Siston R.A., Goodman S.B., Delp S.L., Giori N.J. (Oct 2007). Coronal plane stability before and after total knee arthroplasty. Clin Orthop Relat Res.

[31] Matsumoto T., Muratsu H., Kubo S., Matsushita T., Kurosaka M., Kuroda R. (Oct 2012). Intraoperative soft tissue balance reflects minimum 5-year midterm outcomes in cruciate-retaining and posterior-stabilized total knee arthroplasty. J Arthroplasty.

[32] McAuliffe M.J., O'Connor P.B., Major L.J., Garg G., Whitehouse S.L., Crawford R.W. (Mar 2020). Highly satisfied total knee arthroplasty patients display a wide range of soft tissue balance. J Knee Surg.

